# Supplementary material for: Complexome profiling of the Chlamydomonas psb28 mutant reveals TEF5 as an early PSII assembly factor
Source: Plant Cell. 2025 Mar 17;37(6):koaf055. doi: 10.1093/plcell/koaf055 (PMC12164590; doi:10.1093/plcell/koaf055)
Supplement: koaf055_Supplementary_Data [file koaf055_supplementary_data.zip › psb28_interactive.zip!/plots/intensity/qualityPlots/peptides/_Cre16.g649300.t1.2.html]

### Cre16.g649300.t1.2

Trivial name:   
SeqCoverage [median]: 30.90  
Mass: 25.14 kDa  
  
 

| Sequence | MissedCleavage | Mass [Da] | wtIntensity | muIntensity | Score |
| --- | --- | --- | --- | --- | --- |
| DAGAIRR | 1 | 757.42 | 346.07 | 637.92 | 97.07 |
| LRDVVDVNK | 1 | 1056.59 | 0.00 | 431.83 | 53.12 |
| RALEVLGGGAGGSPFAELDMR | 1 | 2102.06 | 0.00 | 775.32 | 60.94 |
| SAIHPTAMLAAAADDEVAAAAAAER | 0 | 2393.16 | 0.00 | 1864.91 | 88.22 |
| VSEAVRPQQLR | 1 | 1281.72 | 1442.99 | 3725.91 | 69.98 |
|  |  |  | 1789.1 | 7435.9 |  |
